# Systematic Review and Meta-Analysis of Genome-Wide CRISPR-Cas Screens to Identify Host Factors Involved in HIV-1 Infection

**DOI:** 10.3390/v18101135

**Published:** 2026-10-13

**Authors:** Min Zhu, Zhou Sun, Ke Xu, Ling Ye

**Affiliations:** 1 Department of HIV/AIDS Control and Prevention, Hangzhou Center for Disease Control and Prevention, Hangzhou Health Supervision Institution, Hangzhou 310021, China; zm0118@ustc.edu.cn (M.Z.);; 2 Zhejiang Key Laboratory of Multi-Omics in Infection and Immunity, Hangzhou 310021, China

**Keywords:** HIV-1, host factors, CRISPR-Cas9, genome-wide screen, meta-analysis

## Abstract

Human immunodeficiency virus type 1 (HIV-1) replication depends on a complex interplay between viral proteins and host cellular factors, which represent promising therapeutic targets. However, systematic integration of results from genome-wide CRISPR screens for HIV host factors remains limited. Here, we performed a systematic review and meta-analysis of pooled genome-wide CRISPR-Cas9 screens to identify host dependency, restriction, and latency-promoting factors in HIV-1 infection. Following PRISMA guidelines, we identified 11 eligible studies from which we extracted 26 gene lists. These were classified into three phenotypic categories, including dependency (5 lists), restriction (10 lists), and latency-promoting (7 lists), and integrated using meta-analysis by information content (MAIC). GO over-representation analysis and Gene Set Enrichment Analysis were performed on MAIC-ranked gene lists to characterize pathway architectures. MAIC successfully recovered well-established host factors, including the entry receptors CD4, CCR5, and CXCR4; the integration cofactor PSIP1 (LEDGF/p75); and the restriction factors TRIM5, TRIM22, PML, G3BP1, and EIF2AK2. RNF114 emerged as the top-ranked restriction factor, supported by four independent publication-level categories. Additional high-confidence hits included TRIM56, BIRC3, DTX3L, and components of the ubiquitin–proteasome system across the dependency and latency datasets, further supporting the robustness of the MAIC framework. Beyond these canonical factors, we identified multiple undercharacterized genes and pathways, including coagulation and IL-17 signaling in dependency factors, SUMOylation and DNA damage response in restriction factors, and the Integrator complex and proteasome-granule co-enrichment in latency-promoting factors. Leading-edge analysis confirmed that most of these non-canonical enrichments are driven by coherent gene modules rather than a small set of pleiotropic hubs. These findings reveal distinct pathway architectures across the three infection states and highlight previously unrecognized host processes that may serve as targets for antiviral therapy. Our analysis provides a comprehensive resource for guiding future mechanistic studies of host-HIV interactions and developing strategies to enhance immunity, block viral replication, and reverse latency.

## 1. Introduction

Human immunodeficiency virus type 1 (HIV-1) infection remains a major global public health challenge. Although combination antiretroviral therapy (ART) effectively suppresses viral replication and greatly reduces HIV-1-related morbidity and mortality, most ART drugs target viral proteins such as reverse transcriptase, protease, and integrase, and cannot eliminate latent proviruses integrated into the host genome [1]. Consequently, treatment interruption leads to rapid viral rebound, requiring lifelong therapy for most people living with HIV [2]. Long-term ART is associated with issues of adherence, toxicity, drug interactions, and emerging resistance, underscoring the need for novel strategies toward a functional cure [3].

The HIV-1 life cycle critically depends on host cellular machinery, with host proteins regulating virtually every step of viral replication, including entry, reverse transcription, nuclear import, integration, transcription, RNA processing, translation, assembly, and release [4,5,6,7,8]. Simultaneously, host cells employ multiple antiviral mechanisms to limit infection, such as innate immune sensing, restriction factor activity, epigenetic regulation, and post-transcriptional control [9,10,11,12,13]. Therefore, systematic identification of host dependency factors (HDFs) that support viral replication, host restriction factors (HRFs) that inhibit infection, and host factors that regulate HIV-1 latency is essential. Elucidating these interactions not only deepens our understanding of HIV-host biology but also reveals new therapeutic targets. Compared with direct-acting antivirals, host-targeted therapies may be less prone to viral mutation and could offer novel approaches to control or reduce the latent reservoir [14,15]. The CCR5 antagonist maraviroc provides an example of this strategy, which blocks the interaction between HIV-1 and the host co-receptor CCR5 and prevents viral entry into target cells [16].

Before the widespread adoption of CRISPR-Cas systems, RNA interference (RNAi) was the primary method for genome-wide screening of host factors. Early RNAi screens identified numerous candidate genes involved in HIV-1 infection, but the overlap between studies was modest due to incomplete gene knockdown, off-target effects, differences in cell models, and variable screening conditions. These limitations compromise the reproducibility and reliability of RNAi-based results [17,18,19]. CRISPR-Cas9 offers a more direct loss-of-function screening approach. A single-guide RNA directs Cas9 to a specific genomic locus, where Cas9 induces a DNA double-strand break [20]. Repair via non-homologous end joining frequently introduces insertions or deletions that disrupt the open reading frame, resulting in gene knockout. Compared with RNAi, CRISPR knockout typically achieves stronger loss-of-function phenotypes. When combined with pooled sgRNA libraries and high-throughput sequencing, this technology enables large-scale, unbiased dissection of host-virus interactions [21,22,23].

In recent years, CRISPR screens have substantially expanded the landscape of HIV-1 host factors. Early genome-wide knockout screens validated known dependency genes like *CD4*, *CCR5*, *TPST2* and *SLC35B2* [23]. Subsequent CRISPR studies have further expanded the repertoire of HIV-1 host factors, identifying both dependency and restriction factors across different cell types and experimental systems. These screens have collectively provided a rich resource for understanding HIV-host interactions, although the overlap between individual studies remains limited.

Despite this progress, current CRISPR screening data for HIV-1 face several limitations. Different studies employ distinct cell types, viral strains, screening protocols, and statistical thresholds, leading to limited overlap among candidate gene lists. Host dependency factors (HDFs), restriction factors (HRFs), and latency-related factors are typically examined in separate experimental systems, hindering direct cross-comparison. Moreover, inconsistent data reporting (ranked lists vs. binary significant hits) makes simple overlap analysis insufficient for fully leveraging the available evidence. A systematic integration method is therefore needed.

Meta-analysis by Information Content (MAIC) is an algorithm designed to integrate ranked and unranked gene lists from heterogeneous datasets, scoring candidates based on the information content of each study. MAIC has been successfully applied to identify host factors for influenza and other viruses [24,25]. Applying MAIC to HIV-1 CRISPR screens can thus overcome the limitations of individual studies and provide a more comprehensive view of HIV-host interactions.

In this study, we systematically collected published CRISPR screening datasets for HIV-1 infection and categorized host genes into HDFs, HRFs, and latency-related factors. Using MAIC, we generated integrated rankings for each category and performed GO over-representation analysis, GSEA, and EnrichmentMap visualization to identify major functional modules. Our goal was to uncover high-confidence host genes and pathways supported by multiple studies, offering a broader perspective on HIV-1 replication, antiviral defense, and latency and potentially revealing new targets for host-directed therapy.

## 2. Materials and Methods

### 2.1. Data Eligibility

**Figure 1:**
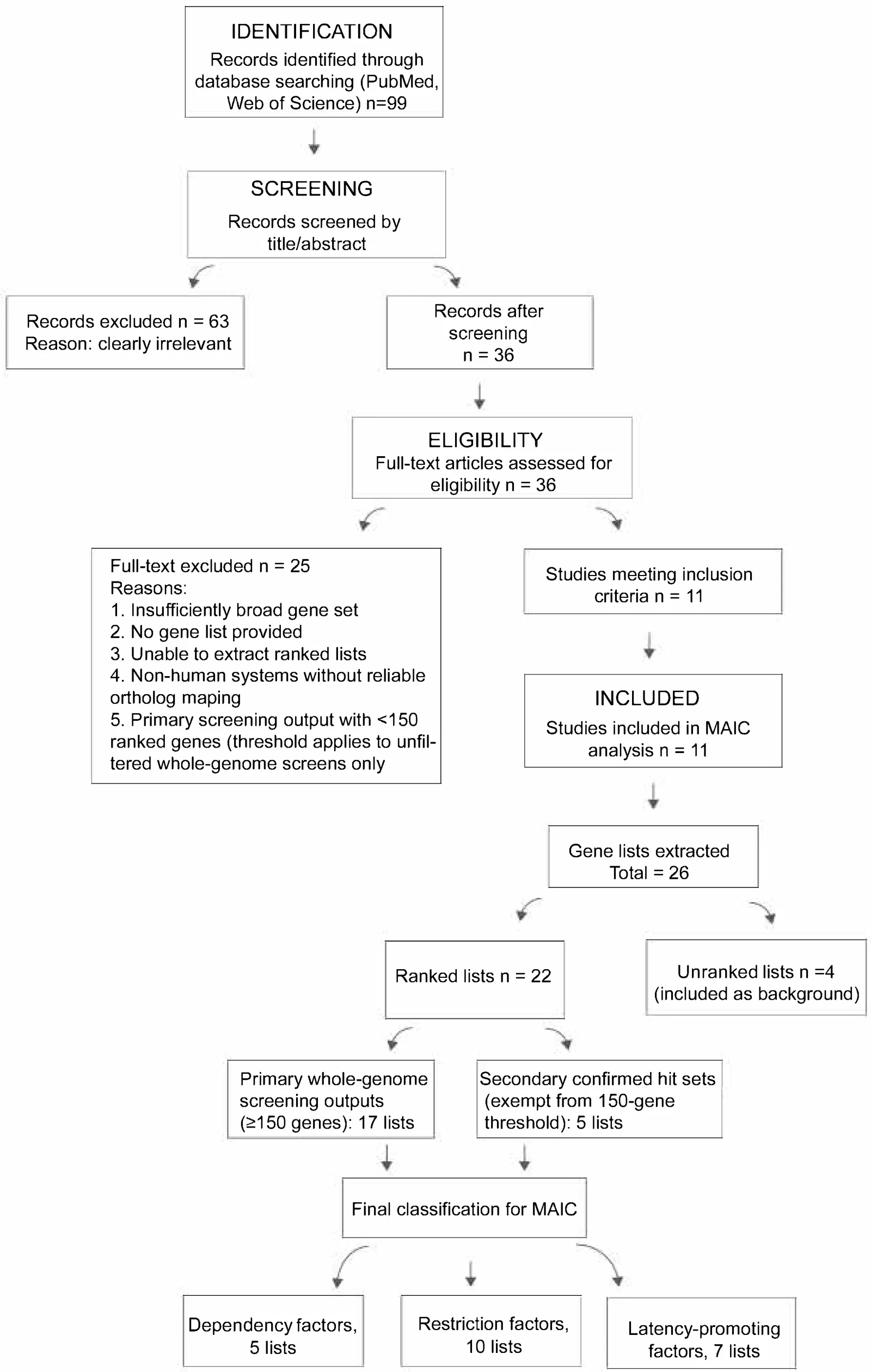
PRISMA flow diagram showing the systematic literature search and study selection process for genome-wide CRISPR screens identifying host factors involved in HIV-1 infection.

Screens were included or excluded based on pre-set inclusion and exclusion criteria, as outlined in Table 1. We included pooled or arrayed genome-wide and targeted CRISPR-Cas screens using replication-competent or single-cycle HIV-1 strains. Screens using pseudotyped viruses that largely consisted of a non-HIV envelope (e.g., VSV-G pseudotyped HIV) were excluded when the gene list was expected to primarily reflect entry pathways of the pseudotype rather than HIV-specific replication, unless the screen also included a wild-type HIV envelope condition for comparison. Screens using an RNAi-based approach (siRNA/shRNA) were excluded because such screens differ fundamentally in their mechanism of gene perturbation and have been shown to produce results with limited overlap with CRISPR-based screens. Screens to identify HIV dependency factors (pro-viral), restriction factors (anti-viral), or latency-promoting factors using cells of human origin, conducted in a loss-of-function (LOF) or gain-of-function (GOF) CRISPR-Cas setting (CRISPR knockout, CRISPR interference [CRISPRi], or CRISPR activation [CRISPRa]), were included. In cases where gene lists of at least 150 genes or raw data (including relevant samples and sgRNA barcodes or gene-level ranked lists) were not available, the screens were excluded. Primary whole-genome screening outputs were required to contain at least 150 ranked genes. However, gene lists derived from secondary validation or confirmation experiments, regardless of list length, were included if they reported ranked validated hits supported by multiple independent replicates or statistical tests. These validation-derived lists are noted separately in Figure 1 and Table 1. Studies from peer-reviewed journals were included; preprint publications were considered only if the data were subsequently published in a peer-reviewed venue.

### 2.2. Literature Search and Study Selection

A systematic review based on the guidelines of PRISMA was performed using PubMed (https://pubmed.ncbi.nlm.nih.gov/; accessed on 25 April 2026) and Web of Science (https://www.webofscience.com; accessed on 25 April 2026). Searches were performed on 25 April 2026 and updated weekly until 10 June 2026. Searches were performed using the following search keywords, without date or language restrictions: ((“HIV” OR “HIV-1”) AND (“CRISPR” OR “CRISPR-Cas9” OR “CRISPRi” OR “CRISPRa”) AND (“screen” OR “screening” OR “genome-wide”) AND (“host factor” OR “dependency factor” OR “restriction factor” OR “latency”)). All returned papers were first assessed for duplication and relevance based on title and abstract. Subsequently, full-length texts were obtained to confirm eligibility according to Table 1. The literature search, screening process, and reasons for exclusion are summarized in a PRISMA flow diagram (Figure 1). Data from included studies were independently extracted by the two reviewers using a standardized form.

### 2.3. Gene List Extraction and Preparation for MAIC

Data relevant to the MAIC analysis and other screen-specific parameters were extracted from eligible studies, and the screens were assigned to one of three groups depending on their experimental setup: dependency factors (pro-viral), restriction factors (anti-viral), or latency-promoting factors. Screens were considered to be ranked when the gene list originated from a gene-ranking analysis (e.g., based on log2 fold change, MAGeCK score, STARS score, enrichment fold, or statistical significance), or otherwise unranked. For screens that provided sgRNA-level data (e.g., enrichment folds for individual sgRNAs), genes were summarized by taking the maximum enrichment value among all sgRNAs targeting the same gene to generate a gene-level ranked list. Non-human genes were mapped to their human homologs using BioMart (Ensembl) (https://e116.ensembl.org; accessed on 12 May 2026) or excluded from the analysis if no human homolog could be identified. According to MAIC requirements [25,26], gene lists were limited to the first 2000 genes when a longer list was provided.

### 2.4. Meta-Analysis by Information Content

MAIC (Meta-analysis by Information Content) was used to integrate heterogeneous gene lists derived from multiple CRISPR screening studies as previously described in MAIC-based integrative analyses of pooled CRISPR screens [25,26]. The approach is designed to combine partially overlapping gene sets originating from sources with variable quality and experimental heterogeneity, without requiring predefined thresholds for significance. As demonstrated in prior MAIC-based meta-analyses of influenza A virus host factors, integration of heterogeneous CRISPR screens successfully identifies high-confidence hits that individual screens fail to rank consistently. Category-based weighting within MAIC ensures that genes supported by multiple independent biological categories receive higher scores than those supported by multiple lists within the same category, effectively mitigating modality-specific biases. In this study, input datasets consisted of gene lists *L*_1_, *L*_2_, …, *L_m_*, each containing a ranked or unranked set of genes, and were assigned to *K* biologically defined categories *C*_1_, *C*_2_, …, *C_K_*. The primary categorization for MAIC weighting was defined at the publication level: gene lists derived from the same publication were grouped into a single category to avoid redundancy from closely related experimental designs [25]. While additional annotations, including cell type, viral strain, and screening design, are provided in Table 1 for descriptive purposes, these were not used as independent weighting categories. Each gene was assigned an initial score based on its occurrence across lists and, where available, its rank position within each list. For ranked datasets, higher-ranked genes contributed more strongly, modeled using an exponential decay weighting function applied to normalized rank positions. Gene scores were iteratively updated based on cross-list and cross-category evidence, such that genes supported by multiple independent categories received higher weights than those supported by multiple lists within the same category. Each gene list was then assigned a weight *w_L_*, defined as the sum of gene scores contained in that list. List weights and gene scores were updated iteratively until convergence, defined as a maximum change in list weights below 0.01 between successive iterations. The final MAIC score for each gene was obtained from the converged model, and genes were ranked in descending order of their MAIC scores. This procedure prioritizes genes supported by consistent evidence across independent biological categories while down-weighting redundancy from highly similar datasets. The custom R scripts (R version 4.5.3) for the MAIC analysis are openly available in the GitHub repository: https://github.com/Min123-xu/HIV-MAIC-host-factors (accessed on 28 July 2026).

### 2.5. Analysis and Visualization of MAIC Inputs and Results

The consistency and complementarity among individual CRISPR screens were first evaluated prior to MAIC. UpSet plots (UpSetR version 1.4.1) were generated to visualise the intersections of the top 2000 genes from each list for restriction, dependency, and latency-promoting factors, with list sizes indicated by left horizontal bars and intersection sizes by vertical bars above the matrix (only the 30 largest intersections are shown). Lists were truncated to 2000 genes to focus on high-confidence candidates while ensuring comparability across screens. To assess concordance at the publication level, Jaccard similarity coefficients between publication-level categories were calculated based on the ranked gene sets included in the MAIC analysis; related lists from the same publication were assigned to a common category to avoid repeated weighting of closely related datasets, and the resulting similarity matrices were displayed as heatmaps, with darker blue indicating greater overlap. To visualise the MAIC output, heatmaps of the top 50 MAIC-ranked genes for dependency, restriction, and latency-promoting factors were generated, respectively, showing the distribution of top-ranked genes across publication-level categories; within each category, the displayed value represents the maximum normalised rank weight observed among related screens from the same publication, with darker blue indicating a higher relative rank weight and white indicating absence. Finally, the frequency distribution of genes supported across independent categories was plotted using bar plots showing the numbers of genes supported by exactly the indicated number of categories, overlaid with cumulative line plots showing the numbers of genes supported by at least the indicated number of categories, with cumulative counts annotated above the line points. All analyses were performed in R version 4.5.3. Heatmaps and bar plots were using ggplot2 version 4.0.3.

### 2.6. Over-Representation Analysis and Gene Set Enrichment Analysis

To functionally characterize the MAIC-ranked gene lists, over-representation analysis (ORA) and Gene Set Enrichment Analysis (GSEA) were performed.

For ORA, the top 300 genes from each ranked MAIC output (dependency, restriction, and latency-promoting factors) were selected for functional enrichment analysis. Gene symbols were converted to Entrez IDs using the org.Hs.eg.db annotation package (version 3.23.1); unmapped symbols were excluded from further analysis. ORA was performed using the enrichGO function from the clusterProfiler R package (version 4.18.4) against the Gene Ontology (GO) databases (http://geneontology.org; accessed on 12 June 2026), covering biological processes (BP), cellular components (CC), and molecular functions (MF). The human genome was used as the background set. Significantly enriched terms were defined at an adjusted *p*-value threshold of 0.05.

For GSEA, the complete ranked gene lists (all genes ranked by MAIC score) were analysed using the clusterProfiler R package (version 4.18.4) against the KEGG (https://www.genome.jp/kegg/; accessed on 12 June 2026), Gene Ontology (http://geneontology.org; accessed on 12 June 2026), and WikiPathways databases (https://www.wikipathways.org; accessed on 12 June 2026), with significance determined at an adjusted *p*-value threshold of 0.05. Enrichment scores were calculated based on gene rankings derived from MAIC scores. All statistical analyses were performed in R version 4.5.3 with Bioconductor version 3.22.

## 3. Results

### 3.1. Systematic Literature Review and Study Selection

To comprehensively identify host factors that influence HIV-1 infection, we performed a systematic literature search for CRISPR-based genetic screens. The search strategy, detailed in Section 2, yielded 99 studies. Titles and abstracts were screened against predefined eligibility criteria, leading to the exclusion of 63 studies that were clearly irrelevant. The remaining 36 articles were retrieved for full-text assessment. After detailed evaluation, 25 studies were excluded for reasons including: lack of a sufficiently broad gene set, absence of a gene list, experimental designs that did not permit extraction of ranked gene lists suitable for meta-analysis, or use of non-human experimental systems without reliable mapping to human orthologs. Ultimately, 11 studies met all inclusion criteria. From these 11 publications we extracted a total of 26 gene lists, each representing a distinct screening condition or analysis.

The 22 ranked gene lists were classified into three broad phenotypic categories: dependency factors, defined as genes whose knockout reduced HIV-1 infection (five lists); restriction factors, defined as genes whose knockout increased HIV-1 infection (10 lists); and latency-promoting factors, defined as genes whose knockout induced the reactivation of latent HIV-1 (seven lists). The remaining four unranked lists were included as background. Detailed information for each gene list, including the cell type, viral strain, treatment condition, screening approach, ranking method, and number of genes, is provided in Table 2.

### 3.2. Overlap Among Ranked Gene Lists

**Figure 2:**
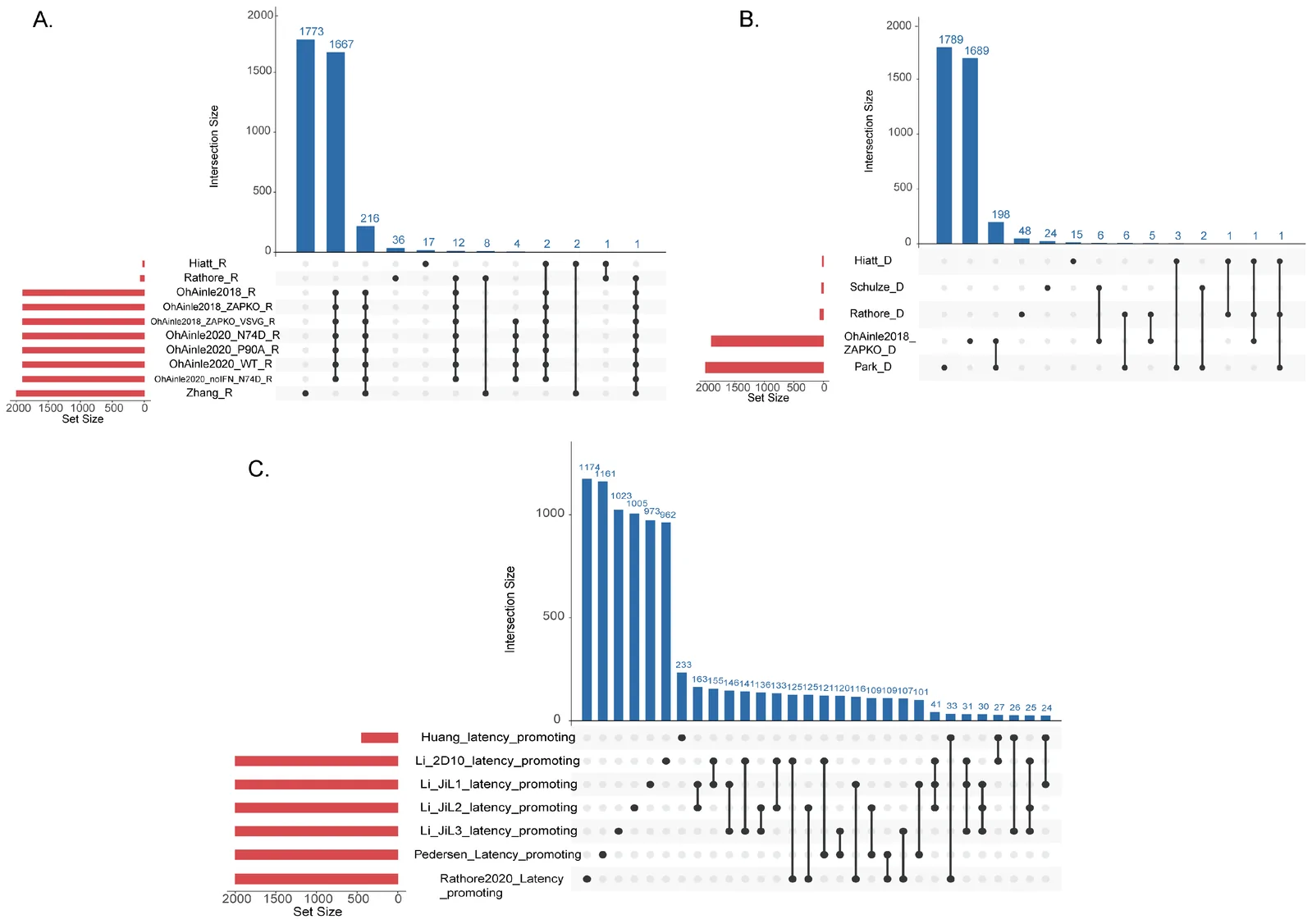
Overlap of top-ranked genes among CRISPR screens for HIV host factors. Upset plots display the intersections of the top 2000 genes from each gene list for (**A**) restriction factors, (**B**) dependency factors, and (**C**) latency-promoting factors. Left horizontal bars show the total number of genes per list. Vertical bars above the matrix indicate the size of each intersection. Only the 30 largest intersections are shown. Lists were truncated to 2000 genes to focus on high-confidence candidates while ensuring comparability across screens of different scales.

To assess the level of agreement among the ranked gene lists, we examined pairwise overlaps among the top-ranked genes reported by the original studies (Figure 2A–C; Supplementary Table S1). To retain high-confidence candidates and reduce noise from genes with low rankings, each list was limited to the top 2000 genes before the overlap analysis and UpSet plot generation. The same cutoff was also applied when preparing the input data for the subsequent MAIC analysis.

Several studies contributed more than one gene list because they included different experimental conditions. OhAinle et al. (2018), for example, provided three lists derived from wild-type THP-1 cells, ZAP-knockout cells, and infection with a VSV-G-pseudotyped virus [28]. OhAinle et al. (2020) provided four lists corresponding to wild-type virus, the P90A mutant, the N74D mutant under interferon treatment, and the N74D mutant without interferon treatment [32]. Li et al. (2020) also provided four lists obtained from the JiL-1, JiL-2, JiL-3, and 2D10 latency models [34]. Gene lists from the same study generally showed greater overlap than those from different studies, likely because they shared experimental platforms, cell models, and analytical procedures. This high within-study similarity raised the possibility that studies contributing several related lists could receive disproportionate weight in the meta-analysis. To address this, we implemented a study-level categorization strategy prior to MAIC, as described in the next section.

Genes detected in several screens included factors involved in NF-κB signalling, P-TEFb-dependent transcription elongation, and the ubiquitin–proteasome system. However, the studies differed considerably in their screening designs, hit-selection criteria, gene-list lengths, and ranking methods. Their gene rankings were therefore not directly comparable. A simple intersection analysis could identify repeatedly detected genes but could not fully use the ranking information or account for differences among datasets. We therefore applied meta-analysis by information content (MAIC), which integrates both ranked and unranked gene lists from heterogeneous sources and generates a global evidence-based ranking of candidate genes.

### 3.3. MAIC Analysis of Included Screens

MAIC was applied separately to the dependency, restriction, and latency-promoting gene lists. All eligible ranked lists were retained. For MAIC weighting, related lists from the same publication were grouped into a single publication-level category to avoid redundancy from closely related experimental designs. During MAIC iteration, for each gene, only the largest contribution among lists within the same category was used, and contributions from different categories were summed. This approach preserved information from distinct experimental conditions while preventing repeated weighting of closely related datasets. Lists from independent studies were treated as separate categories, even when produced by the same research group.

**Figure 3:**
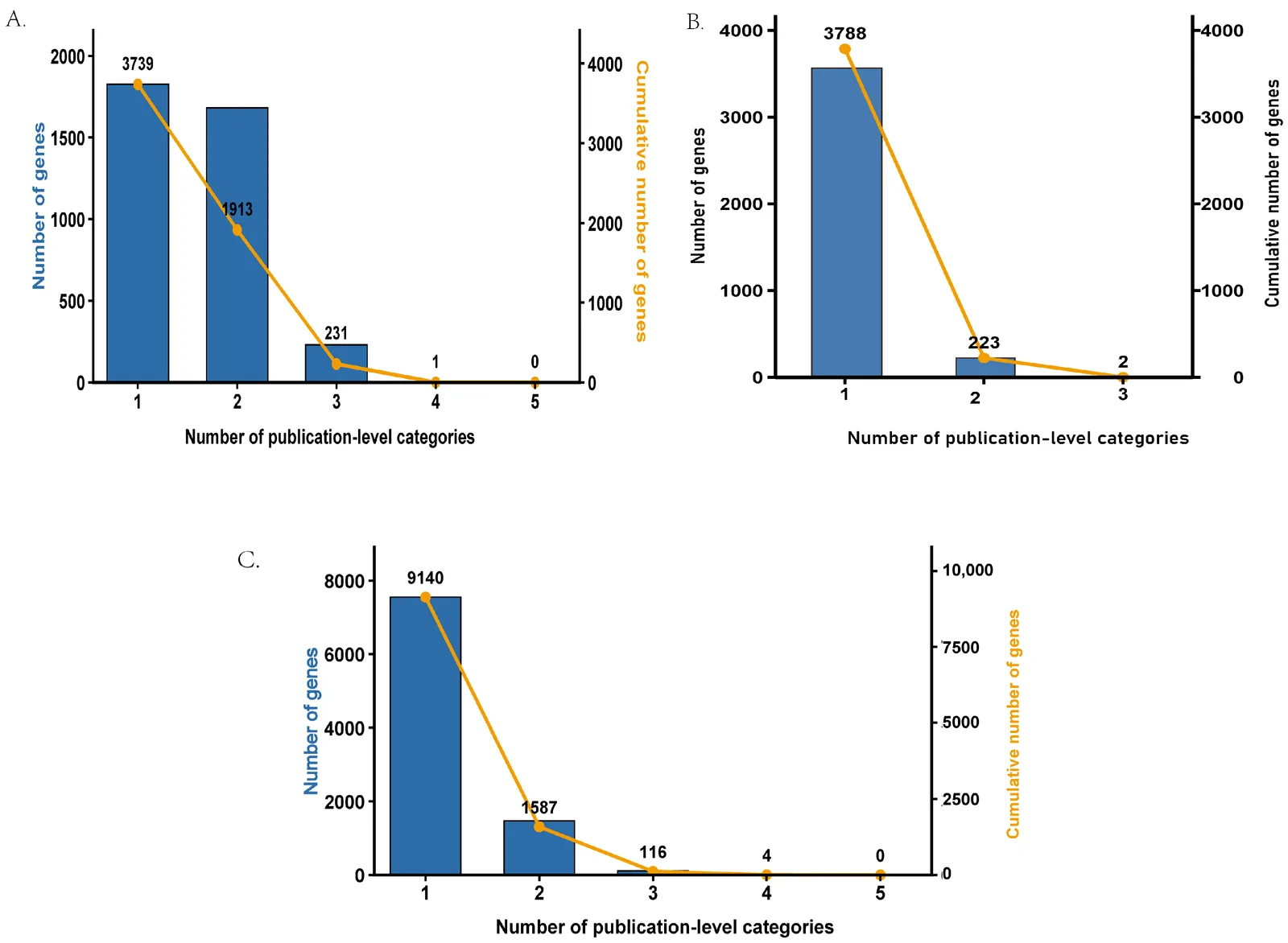
Frequency distribution of host factors supported across independent publication-level categories. Bar plots show the numbers of (**A**) restriction factors, (**B**) dependency factors, and (**C**) latency-promoting factors supported by exactly the indicated number of publication-level categories. The cumulative lines show the numbers of genes supported by at least the indicated number of categories, and the corresponding cumulative counts are annotated above the line points. Related gene lists from the same publication were assigned to a common category to prevent repeated weighting of closely related datasets. The dependency and restriction factor analyses each included five publication-level categories, whereas the latency-promoting factor analysis included four categories. Detailed gene lists are provided in Supplementary Tables S2–S4.

The restriction factor analysis included ten ranked gene lists assigned to five publication-level categories. The three lists from OhAinle 2018 were assigned to one category, whereas the four lists from OhAinle 2020 were assigned to a separate category. The analysis comprised 3739 unique genes, of which 1913 were supported by at least two independent categories. Specifically, 1682 genes were supported by two categories, 230 genes were supported by three categories, and *RNF114* was the only gene supported by four categories. No gene was supported by all five categories. *RNF114* received the highest MAIC score. Other high-ranking genes supported by three categories included *TRIM5*, *IRF2BPL*, *TRIM22*, *BIRC3*, *DTX3L*, *NFXL1*, *TRIM56*, *RNF213*, *TRIM38*, *RBCK1*, *PML*, *ZNRF2*, *GCNT1*, *G3BP1*, *GOSR1*, and *EIF2AK2* (Figure 3A; Supplementary Table S2). These genes were mainly associated with intrinsic antiviral restriction, interferon responses, NF-κB signalling, and ubiquitin-dependent protein regulation.

We assessed the sensitivity of the restriction factor ranking to the inclusion of the CRISPRa dataset [31]. After excluding this GOF (gain-of-function) screen and re-running MAIC with only LOF (loss-of-function) screens (CRISPRko and CRISPRi), 13 of the top 15 restriction factors remained within the top 15, including all canonical factors (TRIM5, TRIM22, PML, G3BP1, EIF2AK2; Supplementary Table S5). Spearman’s rank correlation for the top 50 genes was 0.89. However, several genes dropped substantially in rank (e.g., GOSR1 from 16 to >300), suggesting CRISPRa-specific gain-of-function support. Conversely, interferon-stimulated genes (*STAT1*, *STAT2*, *IFNAR1*, *IRF9*, *MX2*) rose into the top 25, indicating stronger LOF-modal consensus for inducible restriction pathways.

The dependency factor analysis included five ranked gene lists from five independent publication-level categories and comprised 3788 unique genes. Among these, 223 genes were supported by at least two independent categories, including 221 genes supported by two categories and two genes supported by three categories. *CXCR4* and *PSIP1* were the only genes supported by three independent categories. *CXCR4* was identified in the Hiatt, OhAinle 2018, and Rathore datasets, whereas *PSIP1* was identified in the Hiatt, Park, and Rathore datasets. Representative genes supported by two categories included *CD4*, *ELOB*, *CCNT1*, *UHRF1*, *JUNB*, *MYCBP2*, *GLG1*, *ZNF598*, *RNF126*, *TRIM25*, *ALCAM*, *CCR5*, and *SEC22B* (Figure 3B; Supplementary Table S3).

The latency-promoting factor analysis included seven ranked gene lists assigned to four publication-level categories and comprised 9140 unique genes. The four latency models reported by Li (2D10, JiL-1, JiL-2, and JiL-3) [34] were assigned to the same category to avoid repeated weighting of related datasets. Among the 9140 genes, 1587 were supported by at least two independent categories. Specifically, 1471 genes were supported by two categories, 112 genes were supported by three categories, and four genes (*RPS19BP1*, *UBE3C*, *ENC1*, and *HSF4*) were supported by all four categories. Representative genes supported by three categories included *EXOSC8*, *PSMD1*, *PYGO1*, *PSMA5*, *MBNL1*, *BRF1*, *PARPBP*, *BAZ1B*, *DMBX1*, *PCGF1,* and *PDE2A* (Figure 3C; Supplementary Table S4).

### 3.4. Jaccard Similarity and Distribution of Top-Ranked Host Factors

**Figure 4:**
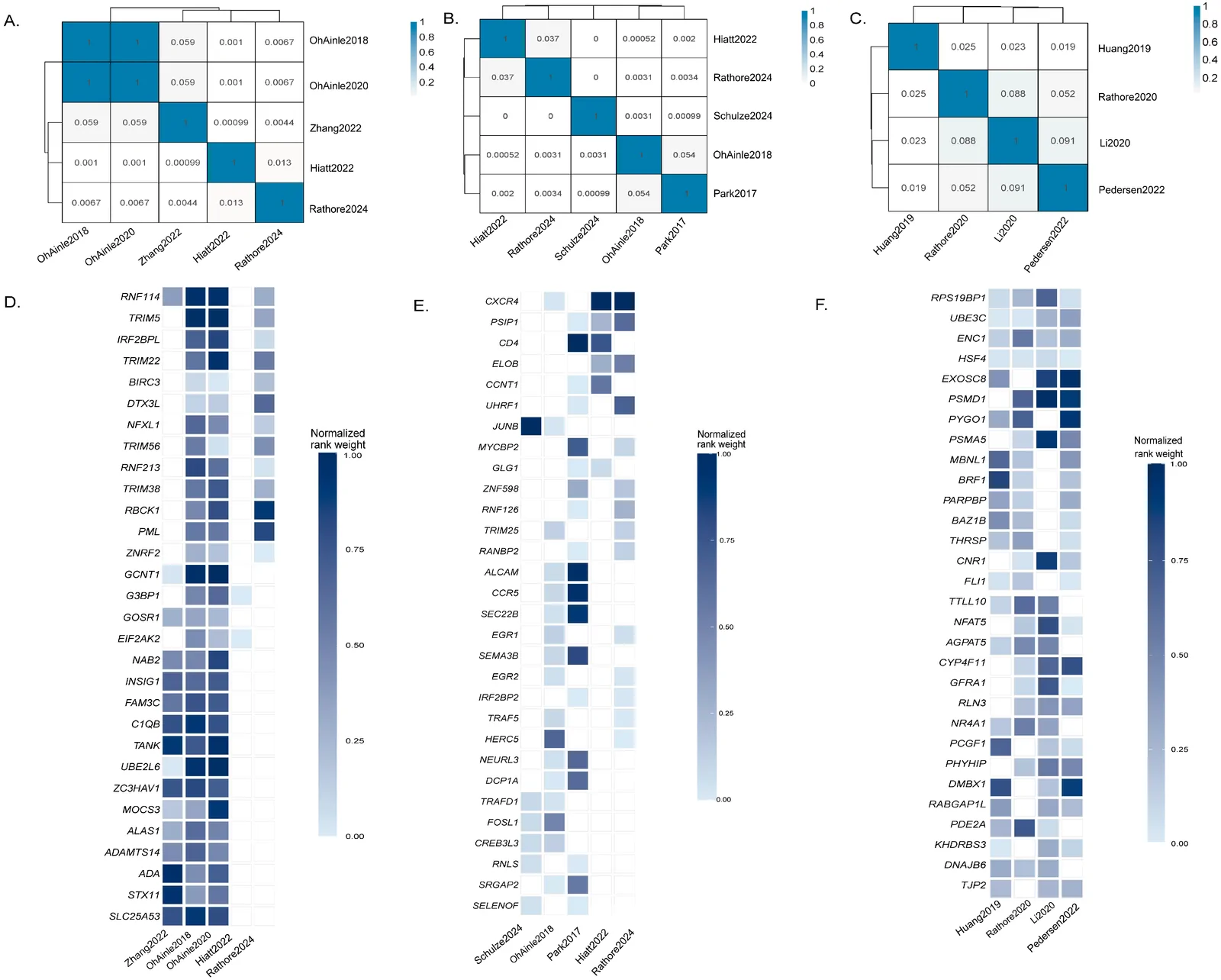
Jaccard similarity and MAIC-derived top-ranked host factors across publication-level categories [23,27,28,29,30,31,32,33,34,35,36]. (**A**–**C**) Jaccard similarity heatmaps for restriction factors (**A**), dependency factors (**B**), and latency-promoting factors (**C**). Each cell shows the Jaccard similarity coefficient between two publication-level categories based on the ranked gene sets included in the MAIC analysis. Darker red indicates greater overlap, whereas lighter colours indicate lower similarity. Related gene lists from the same publication were assigned to a common category to reduce repeated weighting of closely related datasets. (**D**–**F**) Heatmaps of the top 30 MAIC-ranked genes for restriction factors (**D**), dependency factors (**E**), and latency-promoting factors (**F**) across publication-level categories. Within each category, the displayed value represents the maximum normalized rank weight observed among related screens from the same publication. Darker blue indicates a higher relative rank weight, whereas white indicates that the gene was absent from the corresponding category. The dependency-factor heatmap highlights host factors involved in HIV-1 entry, integration, and transcription; the restriction-factor heatmap highlights genes associated with intrinsic antiviral defence, interferon-related responses, and ubiquitin-dependent regulation; and the latency-promoting heatmap highlights genes related to RNA processing, proteasome-mediated protein turnover, transcriptional regulation, and chromatin control.

Jaccard similarity analysis revealed distinct levels of agreement among the three groups of CRISPR screens. The analysis was performed at the publication-category level to reduce the influence of multiple closely related lists from the same study. The five dependency-factor categories showed generally low similarity, indicating substantial heterogeneity among the identified gene sets (Figure 4B). This result was consistent with differences in cell type, viral strain, screening strategy, and hit-selection criteria across studies. In contrast, the restriction-factor datasets showed greater cross-category similarity, with the two OhAinle publication categories displaying particularly strong overlap (Figure 4A). The four latency-promoting categories showed an intermediate pattern, with a shared core signal accompanied by considerable category-specific variation (Figure 4C). These results indicate that experimental context strongly influenced the host factors recovered by individual CRISPR screens.

All eligible lists were retained in the MAIC analysis. Closely related lists from the same publication were assigned to a common publication-level category. For each gene, only the largest normalized rank weight among related lists within the same category was used, whereas evidence from different categories was combined. This approach preserved information from biologically distinct experimental conditions while preventing repeated weighting of closely related datasets.

The restriction factor analysis identified a broad set of candidate genes enriched for functions related to intrinsic antiviral defence and protein homeostasis. *RNF114* achieved the highest MAIC score and was supported by four independent publication-level categories. Other highly ranked genes included *TRIM5*, *TRIM22*, *TRIM38*, *TRIM56*, *RNF213*, *PML*, *DTX3L*, *BIRC3*, *RBCK1*, *NFXL1*, *ZNRF2*, *G3BP1*, *EIF2AK2*, and *GCNT1* (Figure 4D). Well-characterized restriction genes among the top hits included *RNF114*, *TRIM5*, *TRIM22*, *TRIM56*, *G3BP1*, and *EIF2AK2*, all of which have established roles in HIV-1 capsid recognition, transcriptional repression, RNA sequestration, or translational inhibition [37,38,39,40]. Conversely, several high-ranking genes supported by multiple categories remain largely unexplored in the HIV context. These include *IRF2BPL*, *NFXL1*, *RNF213*, *TRIM38*, *ZNRF2*, *GCNT1*, and *GOSR1*, which have limited or no functional annotation in HIV infection and may therefore represent novel restriction candidates for further investigation.

The dependency factor analysis identified a concentrated group of well-established HIV-1 host factors. *CXCR4*, *CD4*, and *CCR5* represented genes involved in viral entry, whereas *PSIP1* and *CCNT1* corresponded to proviral integration and Tat-dependent transcriptional elongation, respectively. The high ranking of *ELOB* further supported a role for ubiquitin ligase-associated regulation in HIV-1 replication. Other highly ranked genes included *UHRF1*, *JUNB*, *MYCBP2*, *GLG1*, *ZNF598*, *RNF126*, *TRIM25*, *RANBP2*, *ALCAM*, *SEC22B*, *EGR1*, *EGR2*, and *IRF2BP2* (Figure 4E). Among these, CD4, CCR5, CXCR4, PSIP1 (LEDGF/p75), and CCNT1 are well-established HIV-1 dependency factors with defined roles in viral entry, integration, or transcription [41,42]. By contrast, MYCBP2, GLG1, RNF126, and SEC22B lack direct functional evidence in the viral life cycle. These less-explored genes thus represent promising candidates for future mechanistic studies.

The latency-promoting factor heatmap identified top-ranked candidates involved in RNA regulation, protein degradation, transcription, and chromatin remodeling (Figure 4F). *RPS19BP1*, *UBE3C*, *ENC1*, and *HSF4* emerged as the highest-confidence candidates, supported across multiple lines of evidence. Several genes implicated in RNA processing (*EXOSC8*) and proteasome-mediated protein turnover (*PSMD1*, *PSMA5*, *UBE3C*) were also highly ranked. Among the remaining top candidates, *FLI1* has been shown to trans-activate HIV-1 LTR-driven transcription [43] and PARPBP may be indirectly linked through PARP inhibitor-related effects on the latent reservoir [44]. In contrast, *MBNL1*, *PYGO1*, *CNR1*, and *GFRA1* lack direct functional evidence linking them to HIV-1 latency, viral transcription, or host factor networks, despite their high MAIC scores, and may represent novel candidates for future investigation.

A comprehensive functional annotation and literature status classification for the top 50 restriction, dependency and latency-promoting factors, including gene descriptions and categorization as well-established, understudied, or novel, is provided in Supplementary Tables S2–S4.

### 3.5. GO Enrichment Analysis of MAIC-RANKED Gene Lists

To characterize the functional landscape of host factors across distinct infection states, we performed Gene Ontology (GO) over-representation analysis (ORA) on the top 300 genes from each MAIC-ranked list (dependency, restriction, and latency-promoting factors), as well as Gene Set Enrichment Analysis (GSEA) on the respective full ranked gene lists (3788 dependency factors, 3739 restriction factors, and 9140 latency-promoting factors). As a proof of concept, ORA successfully recapitulated numerous well-established HIV-1 dependency pathways, including those involved in viral entry and co-receptor engagement (e.g., CCR5, CXCR4), nuclear import and integration (e.g., LEDGF/p75, TNPO3, NUP153), NF-κB-mediated transcriptional activation, and proteasomal regulation. Concurrently, canonical antiviral restriction factors and interferon-responsive pathways (e.g., TRIM5α, APOBEC3G, MX2, STAT1/2) were significantly enriched in the restriction factor dataset, confirming the reliability of our analytical framework. Beyond these established pathways, our analysis uncovered several emerging functional modules that are highly ranked by MAIC but remain relatively understudied, revealing a marked functional divergence among the three factor classes.

**Figure 5:**
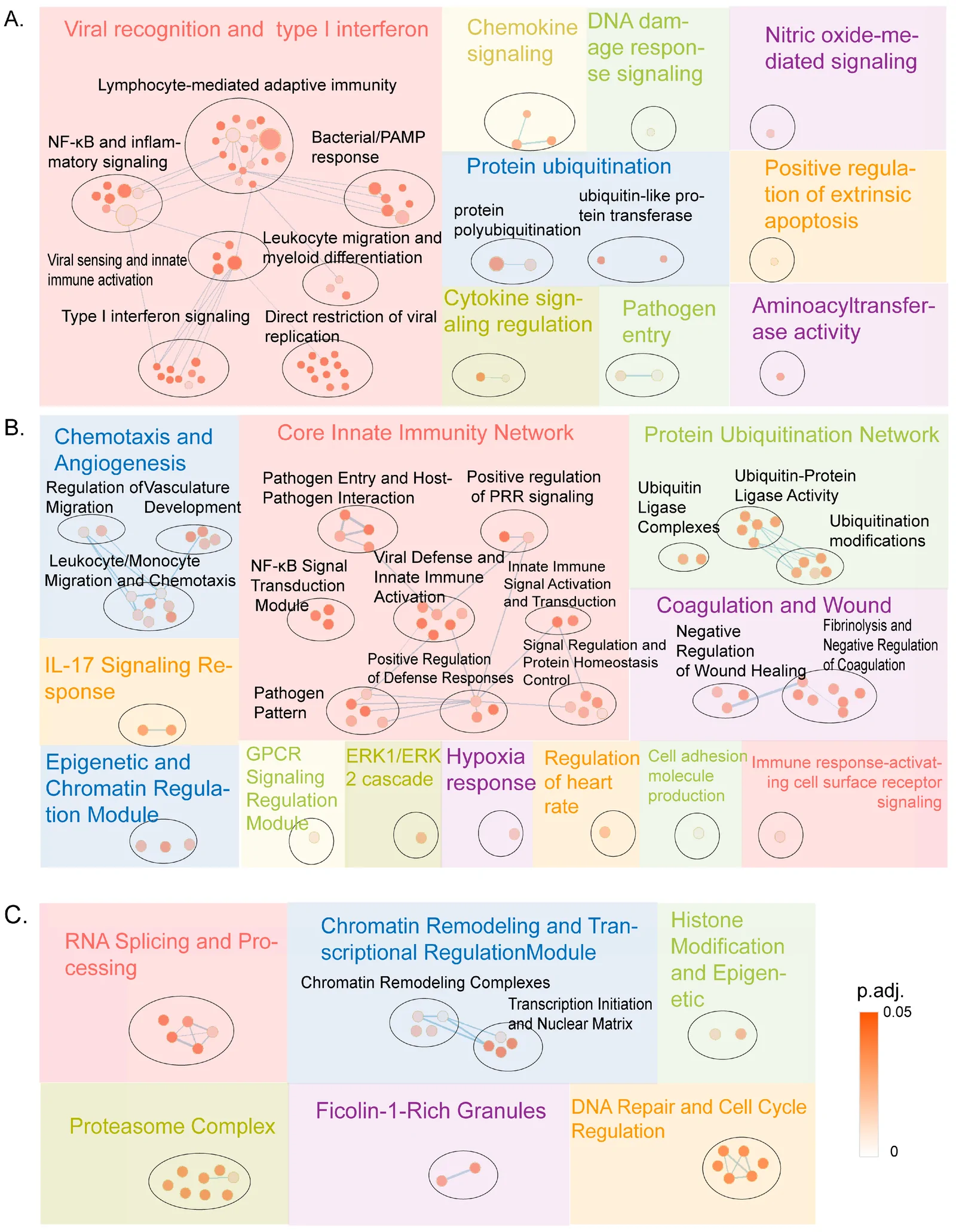
Enrichment Map of GO biological processes enriched among restriction, dependency, and latency-promoting factors. Enriched pathways from clusterProfiler analysis were visualized using EnrichmentMap in Cytoscape version 3.9.1. Nodes represent GO terms, with node color indicating adjusted *p*-value (red: more significant). Edges connect nodes sharing overlapping genes. Clusters were identified by AutoAnnotate and manually annotated into functional modules. (**A**) Restriction factors: five modules including viral sensing/type I IFN, NF-κB/inflammatory signaling, direct viral restriction, immune cell migration/differentiation, and chemokine signaling. (**B**) Dependency factors: innate immune recognition/signaling/defense, leukocyte trafficking/angiogenesis, protein ubiquitination, coagulation/wound healing, and IL-17 signaling. (**C**) Latency-promoting factors: RNA splicing/processing, chromatin remodeling/transcriptional regulation, proteasome complex, DNA repair/cell cycle regulation, and ficolin-1-rich granules.

ORA on the restriction factor dataset revealed approximately 70 enriched pathways (adjusted *p* ≤ 0.05). As anticipated, the most significantly enriched pathways were those well-documented in the antiviral restriction literature, including defense response to virus (GO:0051607, GO:0009615), interferon signaling (GO:0034340, GO:0060337), negative regulation of viral processes (GO:0045071, GO:1903900), NF-κB and inflammatory signaling (GO:0007249, GO:0043122), and cytokine-mediated signaling (GO:0001959, GO:0060759). Beyond these well-characterized antiviral pathways, ORA uncovered several relatively understudied pathways that may be relevant to HIV restriction, including chemokine signaling (GO:0070098, GO:1990868), T-cell and B-cell differentiation and activation (GO:0002285, GO:0030098), and TNF signaling (GO:0033209, GO:0034612). Additionally, enrichment of the DNA damage response pathway (GO:0042770) was observed in the restriction factor dataset. This finding is noteworthy, as DNA damage signaling is not conventionally considered a primary arm of antiviral restriction. Leading-edge analysis revealed that this enrichment is driven by a distributed module of 11 genes, including six dedicated DDR components (*BRIP1*, *BRSK1*, *PPP1R10*, *GML*, *HIC1*, *RAD9A*) alongside established restriction factors such as PML and PARP9 (Supplementary Table S6), arguing against a pleiotropy-driven artifact. Nitric oxide-mediated signal transduction (GO:0007263) was also significantly enriched, although this pathway remains relatively underexplored in the HIV restriction field. We note that this enrichment is based on a small gene set (*AGT*, *NOS2*, *VEGFA*, *APOE*) and should therefore be interpreted with caution until experimentally validated (Supplementary Table S6). Enrichment of extrinsic apoptotic signaling (GO:2001238) suggested that programmed cell death pathways may also contribute to restriction mechanisms. Collectively, these findings suggest that restriction factors may operate through diverse mechanisms beyond classical interferon-stimulated genes, including genome surveillance, DNA repair, and programmed cell death pathways (Figure 5A).

ORA on the dependency factor dataset identified approximately 72 enriched pathways (adjusted *p* ≤ 0.05). As expected, this analysis captured multiple classical pathways known to be exploited by HIV-1 during its replication cycle, including protein ubiquitination (GO:0000209, GO:0070534), pattern recognition receptor signaling (GO:0002221, GO:0002753), NF-κB signal transduction (GO:0007249, GO:0043122), and innate immune activation (GO:0002218, GO:0002758). Additionally, leukocyte migration and chemotaxis pathways (GO:0050900, GO:0030595) and the ERK1/ERK2 cascade (GO:0070371) were significantly enriched, consistent with their established roles in HIV pathogenesis. Notably, ORA also revealed multiple pathways that have not been extensively explored in the context of HIV dependency, including regulation of hemostasis and coagulation (GO:0030195, GO:0050818), wound healing and vascular development (GO:0061045, GO:1903035), and IL-17 signaling (GO:0097396, GO:0097398). Leading-edge gene analysis demonstrated that the hemostasis/coagulation and wound-healing enrichments are driven by a coherent module of seven distinct genes (*PLAT*, *THBD*, *SERPING1*, *SERPINE1*, *KRT1*, *C1QTNF1*, *PDGFA*), five of which are canonical components of the fibrinolytic/coagulation cascade; overlap with wound-healing GOs reflects the well-established biological coupling of coagulation and tissue repair rather than single-gene pleiotropy (Supplementary Table S7). Vasculature development is supported by a broader network of 11-15 distinct genes with minimal overlap with the coagulation module (Supplementary Table S7). While coagulation and fibrinolytic pathways have been clinically associated with HIV infection through the frequent observation of elevated D-dimer and fibrinogen levels as markers of systemic hypercoagulability [45,46], their enrichment in dependency factor screens has not been systematically reported. In addition, IL-17 signaling has been implicated in HIV pathogenesis. Th17 cells are preferentially depleted during HIV infection, which contributes to gut barrier damage and microbial translocation [47]. However, its emergence in dependency-focused screens has received limited attention to date. We caution that the IL-17 enrichment is driven by a small gene set (*TRAF5*, *NFKB1*, *IL1B*, *TRIM32*; n = 4) with substantial pleiotropy, and should be considered preliminary (Supplementary Table S7). Additional enriched pathways included mononuclear cell migration (GO:0071674, GO:0071677, GO:0071675), integrated stress response (GO:0140467), protein SUMOylation (GO:0016925), and epigenetic regulation (GO:0040029). The SUMOylation enrichment is supported by six distinct genes representing different nodes of the SUMO machinery (*RANBP2*, *UHRF2*, *NSMCE1*, *EGR1*, *EGR2*, *IFIH1*), suggesting distributed pathway involvement rather than a single pleiotropic hub (Supplementary Table S7). The identification of these less-explored pathways, particularly those involved in coagulation, wound healing, and IL-17 signaling, suggests that HIV dependency may extend beyond classical immune and viral replication pathways to encompass broader physiological processes. Such processes have been largely overlooked in previous genome-scale screens (Figure 5B).

ORA on the latency-promoting factor dataset revealed a fundamentally different pathway landscape, identifying only approximately 31 enriched pathways (adjusted *p* ≤ 0.05), reflecting the distinct biological nature of viral persistence versus active replication. The most significantly enriched pathways involved RNA splicing and processing (GO:0006397, GO:0008380), chromatin remodeling and transcriptional regulation (GO:0016586, GO:0070603), and the proteasome complex (GO:0000502, GO:0005838). Pathways involved in DNA repair and cell cycle regulation were also significantly enriched (GO:0000724, GO:0098687). Among these, the enrichment of SWI/SNF and RSC chromatin remodeling complexes is particularly noteworthy and is well supported by the literature [48]. The role of the proteasome complex in HIV latency is also well documented. The FDA-approved proteasome inhibitors bortezomib and carfilzomib synergize strongly with existing latency-reversing agents to reactivate HIV-1 in CD4+ T cells from antiretroviral therapy-suppressed individuals [49]. Notably, the enrichment of ficolin-1-rich granule pathways (GO:0101002, GO:1904813) represents a novel finding not previously associated with HIV latency. However, leading-edge analysis revealed that this enrichment is predominantly driven by proteasome subunits (8 of 11 genes: *PSMA5*, *PSMC2*, *PSMD2/3/6/12/13/14*), with only three non-proteasome genes (*ALOX5*, *FGL2*, *PNP*) (Supplementary Table S8). This composition suggests that the enrichment may reflect either GO annotation overlap between the proteasome complex and granule lumen proteome or genuine proteasome co-localization within neutrophil granules, rather than a discrete granule-specific pathway. We have therefore framed this finding as proteasome-granule co-enrichment that warrants further experimental dissection. Beyond the proteasome and ficolin-1-rich granule overlap, the latency-promoting factor pathway map revealed additional undercharacterized modules, including the Integrator complex in snRNA metabolism (INTS3, INTS5, INTS6; GO:0016073), G2/M cell cycle checkpoint regulation (WEE1, RAD17, TOPBP1, PDIK1L, MBTPS1; GO:0010972), and double-strand break repair via homologous recombination (PARPBP, MCMDC2, NSMCE4A, LIG3; GO:0000724). Leading-edge analysis confirmed that these enrichments are supported by distributed gene modules with limited cross-module overlap that reflects known biological coupling (e.g., chromatin remodelers recruited to DNA repair sites) rather than pleiotropic artifacts (Supplementary Table S8). Taken together, these findings strongly suggest that HIV latency is primarily maintained by fundamental cellular processes, including RNA processing, chromatin remodeling, proteasome-mediated protein degradation, and DNA repair, rather than by active immune signaling (Figure 5C).

GSEA on full ranked lists showed similar but more generalized enrichments to ORA, supporting robustness (Supplementary Figures S1 and S2). Collectively, the three factor classes show distinct pathway architectures, with potential targets including coagulation/wound healing in dependency, DNA damage/NO signaling in restriction, and Integrator/proteasome-granule co-enrichment in latency.

## 4. Discussion

In this study, we employed MAIC to integrate multiple genome-wide CRISPR-Cas9 screening datasets and generated ranked gene lists for three functionally distinct categories of host factors, namely restriction factors, dependency factors, and latency-promoting factors. Through systematic GO over-representation analysis and GSEA, we characterized the pathway architectures of these three factor classes, revealing both well-established mechanisms and undercharacterized functional modules that warrant further investigation. Although individual CRISPR screens may be subject to inherent biases, our integrative approach successfully identified host factors and pathways that are potentially involved in distinct stages of HIV infection, ranging from active replication to latent persistence.

The validity of our method is demonstrated by its ability to accurately identify known host factors in the top results. For dependency factors, ORA captured protein ubiquitination, pattern-recognition receptor signaling, NF-κB, and innate immune activation; NF-κB is critical because the HIV LTR contains NF-κB sites. For restriction factors, defense response to virus, interferon signaling, negative regulation of viral processes, and NF-κB/inflammatory signaling were enriched, covering APOBEC3, TRIM5α, Tetherin, and MX2. For latency-promoting factors, SWI/SNF chromatin remodeling, RNA splicing, and the proteasome were identified. Recapitulation of these canonical pathways validates MAIC ranking and provides a foundation for discovery.

In dependency factors, coagulation/hemostasis, wound healing/vascular development, IL-17 signaling, and SUMOylation were less explored. Chronic HIV infection is associated with elevated D-dimer and fibrinogen, and D-dimer predicts morbidity [50]. However, the enrichment of these pathways in dependency factor screens has not been systematically reported. Leading-edge analysis showed that coagulation enrichment was driven by seven genes (*PLAT*, *THBD*, *SERPING1*, *SERPINE1*, *KRT1*, *C1QTNF1*, *PDGFA*), with overlap with wound healing reflecting known biological coupling. HIV-1 p17 activates endothelial cells and may link replication to coagulation dysregulation [51]. *PLAT*, *SERPINE1*, and *THBD* have unclear roles in HIV infection. IL-17 signaling was based on a small, pleiotropic gene set (*TRAF5*, *NFKB1*, *IL1B*, *TRIM32*; n = 4) and is preliminary. SUMOylation has recently gained attention in antiviral signaling [52]. SAMHD1 SUMOylation at Lys595 is required for HIV-1 restriction, and SUMOylated substrates can interact with SIM motifs on TRIM5α, SAMHD1, and Daxx [53]. Our enrichment included *RANBP2* and *NSMCE1*, supporting a distributed SUMOylation module in dependency factors.

For restriction factors, ZC3HAV1 (ZAP) and N4BP1 remain understudied in HIV-1 restriction. DNA damage response (GO:0042770) was enriched through 11 genes, including six dedicated DDR components plus PML and PARP9, suggesting genome surveillance/DNA repair as an underappreciated restriction arm. NO signaling (four genes) and extrinsic apoptosis were also enriched but require caution/validation.

For latency-promoting factors, beyond SWI/SNF and proteasome, several almost unstudied pathways/genes emerged. INTS12 knockdown enhances HIV-1 reactivation [54]. Given the central role of this complex in transcriptional regulation, other subunits, including INTS3, INTS5, and INTS6, may also contribute to proviral transcriptional silencing, warranting further investigation into their potential functions in latency maintenance. Within the cell cycle checkpoint pathway, *WEE1* and *ETAA1* are two particularly noteworthy genes. *WEE1* is a key kinase at the G2/M checkpoint that inhibits cell entry into mitosis through phosphorylation of CDK1 [55]. The potential roles of these cell cycle regulators in HIV latency are completely unknown. Resting CD4+ T cells are the major latent reservoir, so targeting checkpoints may destabilize latency. *RAD17* is another gene of interest. As a component of the DNA damage checkpoint sensor complex, it plays a critical role in DNA damage repair [56]. The HIV integration process itself generates DNA damage signals, and the enrichment of *RAD17* suggests that DNA damage checkpoints may play a role in post-integration transcriptional silencing.

Notably, the enrichment of ficolin-1-rich granule pathways (GO:0101002, GO:1904813) in latency-promoting factors was identified in our analysis. Ficolin-1 is a pattern recognition molecule of the lectin complement pathway, and its potential role in HIV latent infection is entirely unknown. However, leading-edge analysis revealed that this enrichment is predominantly driven by proteasome subunits (8 of 11 genes: *PSMA5*, *PSMC2*, *PSMD2/3/6/12/13/14*), with only three non-proteasome genes (*ALOX5*, *FGL2*, *PNP*) (Supplementary Table S8). We propose two non-mutually exclusive interpretations: (i) GO annotation overlap between the proteasome complex and granule lumen proteome or (ii) genuine proteasome co-localization within neutrophil granules. Regardless of the mechanistic basis, we emphasize that this enrichment is proteasome-driven and have therefore framed it as proteasome-granule co-enrichment rather than a standalone granule-specific pathway. This finding may point to a previously unrecognized connection between proteasome-dependent protein degradation and granule-associated immune processes in latently infected cells, but requires experimental validation in appropriate cell models.

The three factor classes exhibited markedly distinct pathway architectures. Dependency factors were most prominently characterized by ubiquitination, accompanied by PRR signaling, NF-κB, and innate immune activation. Restriction factors represented a composite module of antiviral immunity, ubiquitination, and inflammatory chemotaxis. Latency-promoting factors displayed features of RNA surveillance, proteasome degradation, and chromatin remodeling. This distinction carries biological significance: dependency factors and restriction factors operate in the context of active viral replication and host defense, whereas latency-promoting factors maintain the virus in a state of transcriptional silencing, rendering it largely invisible to the immune system.

Notably, ubiquitination was highly enriched in both dependency and restriction factors, but its functions may be diametrically opposed. In dependency factors, K63-linked ubiquitination promotes viral transcription through activation of signaling pathways such as NF-κB and IRF3. In restriction factors, K48-linked ubiquitination mediates proteasomal degradation of viral proteins, such as HIV-1 Vif-induced degradation of APOBEC3, while K63-linked ubiquitination serves as a signaling platform for innate immune activation. This dual role of ubiquitination underscores its central position in the host-HIV interplay.

Our analysis has several limitations. First, we acknowledge the integration of one CRISPRa screen with nine LOF screens. CRISPRa identifies gain-of-function phenotypes, whereas CRISPRko/CRISPRi identify loss-of-function phenotypes, and these modalities may capture distinct mechanistic classes of restriction factors. While MAIC’s category-based weighting is designed to accommodate heterogeneous data sources [25], and similar integrations have been reported in the field [26], we acknowledge that this mixing may inflate the scores of genes whose restriction activity is only manifest upon overexpression. Sensitivity analysis confirmed that the core restriction factor repertoire (top 15) is robust to CRISPRa exclusion; nevertheless, genes whose ranking depends primarily on the CRISPRa dataset should be interpreted with caution and require orthogonal validation in LOF models. Second, we included several short validation-derived hit sets (e.g., Hiatt, Rathore, Schulze) alongside longer whole-genome primary screening outputs. Although MAIC’s exponential decay model heavily down-weights long-tail genes in extended lists, and its list weighting is based on mean per-gene scores rather than list length, thereby preventing inflated contributions from either short or long lists, and final gene rankings are driven by cross-study consensus rather than any single list, we acknowledge that this heterogeneity represents an inherent feature of the MAIC framework. Third, the use of arbitrary thresholds, such as the top 300 genes for ORA, may exclude relevant genes falling just below the cutoff. The relatively lower number of enriched pathways for latency-promoting factors compared with dependency factors and restriction factors may reflect the greater challenge of studying latency, a highly heterogeneous and dynamic state that is difficult to capture in CRISPR screens. Fourth, we acknowledge the absence of a formal, widely accepted risk-of-bias or quality assessment tool specifically designed for functional genomic screens; although we implemented basic QC measures (e.g., requirement for genome-scale libraries, positive/negative controls, and established analytical pipelines), this limitation should be considered when interpreting our findings.

## 5. Conclusions

In summary, this systematic meta-analysis of genome-wide CRISPR-Cas9 screens, combined with GO ORA and GSEA, provides a comprehensive functional map of host-HIV interactions spanning three distinct infection states. It draws attention to both well-characterized host pathways, including ubiquitination, NF-κB signaling, interferon responses, SWI/SNF chromatin remodeling, and the proteasome, as well as undercharacterized functional modules, including coagulation, IL-17 signaling, SUMOylation, the DNA damage response, the Integrator complex, and proteasome–granule co-enrichment (the latter previously annotated as ficolin-1-rich granules). Through leading-edge gene analysis, we demonstrate that the majority of these non-classical enrichments are supported by distributed gene modules rather than a few pleiotropic genes, with the exception of IL-17 and nitric oxide signaling, which are based on small gene sets and should be considered preliminary until experimentally validated. The gene lists and pathway annotations generated from this meta-analysis may serve as a valuable resource for guiding future studies of host-HIV interactions and identifying novel therapeutic targets for enhancing immunity, blocking replication, and reversing latency.

## Figures and Tables

**Table 1:** Inclusion and exclusion criteria.

Inclusion	Exclusion
Pooled or arrayed CRISPR-Cas screens	RNAi-based screens (siRNA/shRNA)
HIV-1 strains: wild-type or engineered, replication-competent or single-cycle	Pseudotyped viruses (unless with wild-type HIV envelope comparison)
Screens identifying dependency factors (pro-viral), restriction factors (anti-viral), or latency-promoting factors	Screens without a clear host factor classification
LOF or GOF CRISPR-Cas (CRISPRko, CRISPRi, CRISPRa)	Screens with gene lists containing fewer than 150 genes or without available ranked data *
Cells of human origin	Non-human cell lines
Gene lists of ≥150 genes or available ranked sgRNA/gene data *	Studies with unavailable raw data or gene lists
Peer-reviewed publications	Preprints (unless subsequently published)

Note: * Primary whole-genome screening outputs were required to contain ≥150 genes. Gene lists derived from secondary validation or confirmation experiments were exempt from this threshold if they reported ranked validated hits supported by multiple independent replicates or statistical tests.

**Table 2:** Summary of CRISPR-based gene lists included in the MAIC meta-analysis.

Ref.	List_Label	Ranked/Unranked	Cell Type	Virus/Treatment	Phenotype Category	No. of Genes
[27]	Hiatt_D	Ranked (log2FC)	Primary CD4+ T cells	HIV-1 NL4-3 Nef:IRES:GFP (X4)	Dependency	21
[27]	Hiatt_R	Ranked (log2FC)	Primary CD4+ T cells	HIV-1 NL4-3 Nef:IRES:GFP (X4)	Restriction	22
[27]	Hiatt_Toxic	Unranked	Primary CD4+ T cells	HIV-1 NL4-3 Nef:IRES:GFP (X4)	Toxicity	13
[27]	Hiatt_TRUENEG	Unranked	Primary CD4+ T cells	HIV-1 NL4-3 Nef:IRES:GFP (X4)	No phenotype	297
[27]	Hiatt_undetermined	Unranked	Primary CD4+ T cells	HIV-1 NL4-3 Nef:IRES:GFP (X4)	Undetermined	76
[28]	OhAinle2018_R	Ranked (MAGeCK)	THP-1 (monocytic cell line)	HIV-1 LAI (X4-tropic) + IFNα	Restriction	1902
[28]	OhAinle2018_ZAPKO_R	Ranked (MAGeCK)	THP-1 ZAP KO	HIV-1 LAI (X4-tropic) + IFNα	Restriction	1902
[28]	OhAinle2018_ZAPKO_VSVG_R	Ranked (MAGeCK	THP-1 ZAP KO	VSV-G pseudotyped HIV-1 + IFNα	Restriction	1906
[28]	OhAinle2018_ZAPKO_D	Ranked (MAGeCK)	THP-1 ZAP KO	HIV-1 LAI (X4-tropic) + IFNα	Dependency	1902
[29]	Schulze_D	Ranked (enrichment fold)	TZM-GFP (HeLa-derived)	HIV-1 NL4-3 (X4-tropic)	Dependency	32
[23]	Park_D	Ranked (CRISPR gene score)	GXRCas9 (CCRF-CEM-derived T cell line)	HIV-1 JR-CSF (CCR5-tropic)	Dependency	18,387
[30]	Rathore_D	Ranked (avg.LFC)	Primary CD4+ T cells (activated)	HIV-1 NL4-3 Nef:IRES:GFP (X4-tropic)	Dependency	62
[30]	Rathore_R	Ranked (avg.LFC)	Primary CD4+ T cells (activated)	HIV-1 NL4-3 Nef:IRES:GFP (X4-tropic)	Restriction	58
[30]	Rathore_undetermined	Unranked	Primary CD4+ T cells (activated)	HIV-1 NL4-3 Nef:IRES:GFP (X4-tropic)	Undetermined	82
[31]	Zhang_R	Ranked (STARS score)	MT4 (CD4+ T cell line)	HIV-1 NL4-3 Δnef-TK (X4-tropic) + ganciclovir selection	Restriction	2426
[32]	OhAinle2020_WT_R	Ranked (MAGeCK)	THP-1 (monocytic cell line)	HIV-1 LAI WT + IFNα	Restriction	1906
[32]	OhAinle2020_P90A_R	Ranked (MAGeCK)	THP-1 (monocytic cell line)	HIV-1 LAI CA-P90A (CypA-binding deficient) + IFNα	Restriction	1906
[32]	OhAinle2020_N74D_R	Ranked (MAGeCK)	THP-1 (monocytic cell line)	HIV-1 LAI CA-N74D (CPSF6-binding deficient) + IFNα	Restriction	1906
[32]	OhAinle2020_noIFN_N74D_R	Ranked (MAGeCK)	THP-1 (monocytic cell line)	HIV-1 LAI CA-N74D (CPSF6-binding deficient), no IFN	Restriction	1906
[33]	Huang_latency_promoting	Ranked (HiTSelect Fdr)	J-LAT A2 (latent T cell line)	HIV-1 LTR-Tat-IRES-GFP minigenome (latent provirus)	Latency-promoting	446
[34]	Li_JiL1_latency_promoting	Ranked (enrichment fold)	JiL-1 (Jurkat inducible CRISPRi latency cell line; low inducibility)	HIV-1 d2EGFP reporter (single-round, CXCR4-tropic, VSV-G pseudotyped)	Latency-promoting	18,904
[34]	Li_JiL2_latency_promoting	Ranked (enrichment fold)	JiL-2 (Jurkat inducible CRISPRi latency cell line; intermediate inducibility)	HIV-1 d2EGFP reporter	Latency-promoting	18,904
[34]	Li_JiL3_latency_promoting	Ranked (enrichment fold)	JiL-3 (Jurkat inducible CRISPRi latency cell line; high inducibility)	HIV-1 d2EGFP reporter	Latency-promoting	18,904
[34]	Li_2D10_latency_promoting	Ranked (enrichment fold)	2D10-CRISPRi (Jurkat-based latency model with established transcriptional pausing)	HIV-1 d2EGFP reporter	Latency-promoting	18,904
[35]	Pedersen_Latency-promoting	Ranked (MAGeCK)	Jurkat T cell	HIV-1-d6-GFP reporter (full-length NL4-3 with inactivating mutations in gag, pol, vif, vpr, vpu, env, nef; Env replaced by GFP)	Latency-promoting	18,608
[36]	Rathore2020_Latency-promoting	Ranked (MAGeCK)	J-Lat 10.6	HIV-1 GFP reporter (replication-incompetent, env-defective, nef-deleted)	Latency-promoting	18,880

## Data Availability

This systematic review was registered in PROSPERO (CRD420261438705). All gene lists and ranked datasets used in this meta-analysis were extracted from the Supplementary Materials of the original publications. Processed data and MAIC results are available from the corresponding author upon reasonable request. The custom R scripts (R version 4.5.3) for the MAIC analysis are openly available in the GitHub repository: https://github.com/Min123-xu/HIV-MAIC-host-factors (accessed on 28 July 2026).

## Supplementary Materials

The following supporting information can be downloaded at https://www.mdpi.com/article/10.3390/v18101135/s1, Figure S1: Gene Ontology enrichment analysis of restriction, dependency, and latency-promoting factors; Figure S2: Gene set enrichment analysis (GSEA) of restriction, dependency, and latency promoting factors; Table S1: Gene sets and member genes for UpSet plot analysis; Table S2: MAIC-based ranking of HIV-1 restriction factors with MAIC scores, functional annotations and literature curation; Table S3: MAIC-based ranking of HIV-1 dependency factors with MAIC scores, functional annotations and literature curation; Table S4: MAIC-based ranking of HIV-1 latency-promoting factors with MAIC scores, functional annotations and literature curation; Table S5: MAIC-based ranking of HIV-1 restriction factors, listing gene symbols, MAIC scores, and overall ranks after exclusion of the CRISPRa screen; Table S6: Core leading-edge genes driving non-canonical GO enrichments enriched in the Restriction factor dataset; Table S7: Core leading-edge genes driving non-canonical GO enrichments enriched in the dependency factor dataset; Table S8: Core leading-edge genes driving non-canonical GO enrichments enriched in the Latency-Promoting Factor dataset; Table S9: PRISMA 2020 checklist.
