# Supplementary material for: Systematic Review and Meta-Analysis of Genome-Wide CRISPR-Cas Screens to Identify Host Factors Involved in HIV-1 Infection
Source: Viruses. 2026 Oct 13;18(10):1135. doi: 10.3390/v18101135 (PMC13709478; doi:10.3390/v18101135)
Supplement: Supplementary file 1 [file viruses-18-01135-s001.zip › Supplementary Figures.pdf]

Supplementar Figures:

A.

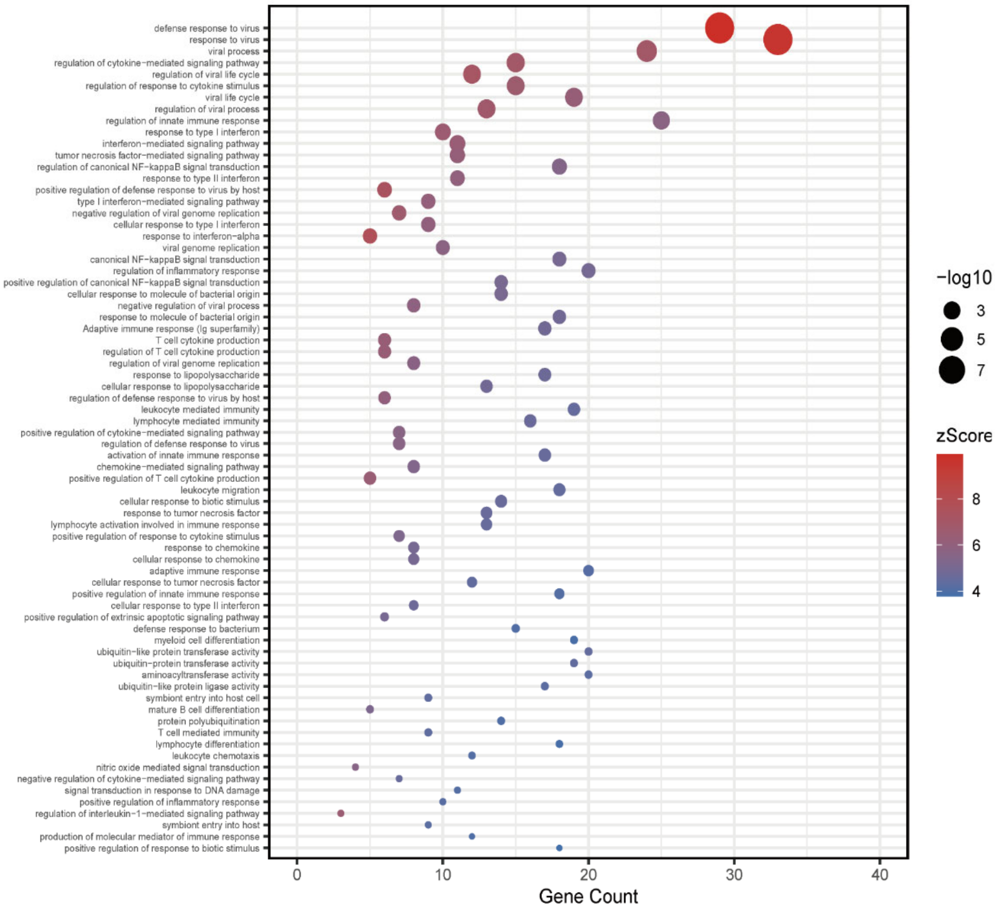

B.

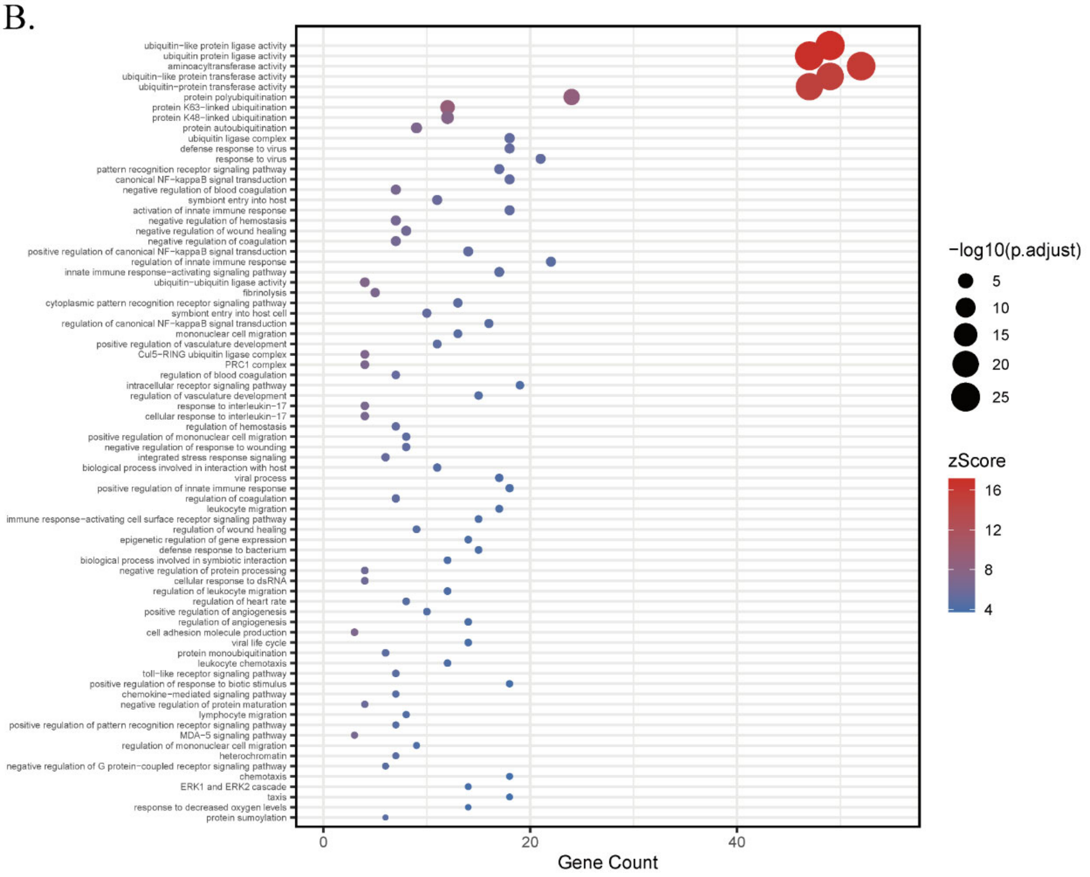

ubiquitin−like protein ligase activity
ubiquitin protein ligase activity
aminoacyltransferase activity
ubiquitin−like protein transferase activity
ubiquitin−protein transferase activity
protein polyubiquitination
protein K63−linked ubiquitination
protein K48−linked ubiquitination
protein autoubiquitination
ubiquitin ligase complex
defense response to virus
response to virus
pattern recognition receptor signaling pathway
canonical NF−kappaB signal transduction
negative regulation of blood coagulation
symbiont entry into host
activation of innate immune response
negative regulation of hemostasis
negative regulation of wound healing
negative regulation of coagulation
positive regulation of canonical NF−kappaB signal transduction
regulation of innate immune response
innate immune response−activating signaling pathway
ubiquitin−ubiquitin ligase activity
fibrinolysis
cytoplasmic pattern recognition receptor signaling pathway
symbiont entry into host cell
regulation of canonical NF−kappaB signal transduction
mononuclear cell migration
positive regulation of vasculature development
Cul5−RING ubiquitin ligase complex
PRC1 complex
regulation of blood coagulation
intracellular receptor signaling pathway
regulation of vasculature development
response to interleukin−17
cellular response to interleukin−17
regulation of hemostasis
positive regulation of mononuclear cell migration
negative regulation of response to wounding
integrated stress response signaling
biological process involved in interaction with host
viral process
positive regulation of innate immune response
regulation of coagulation
leukocyte migration
immune response−activating cell surface receptor signaling pathway
regulation of wound healing
epigenetic regulation of gene expression
defense response to bacterium
biological process involved in symbiotic interaction
negative regulation of protein processing
cellular response to dsRNA
regulation of leukocyte migration
regulation of heart rate
positive regulation of angiogenesis
regulation of angiogenesis
cell adhesion molecule production
viral life cycle
protein monoubiquitination
leukocyte chemotaxis
toll−like receptor signaling pathway
positive regulation of response to biotic stimulus
chemokine−mediated signaling pathway
negative regulation of protein maturation
lymphocyte migration
positive regulation of pattern recognition receptor signaling pathway
MDA−5 signaling pathway
regulation of mononuclear cell migration
heterochromatin
negative regulation of G protein−coupled receptor signaling pathway
chemotaxis
ERK1 and ERK2 cascade
taxis
response to decreased oxygen levels
protein sumoylation
0
20
40
Gene Count
−log10(p.adjust)
5
10
15
20
25
zScore
16
12
8
4

C.

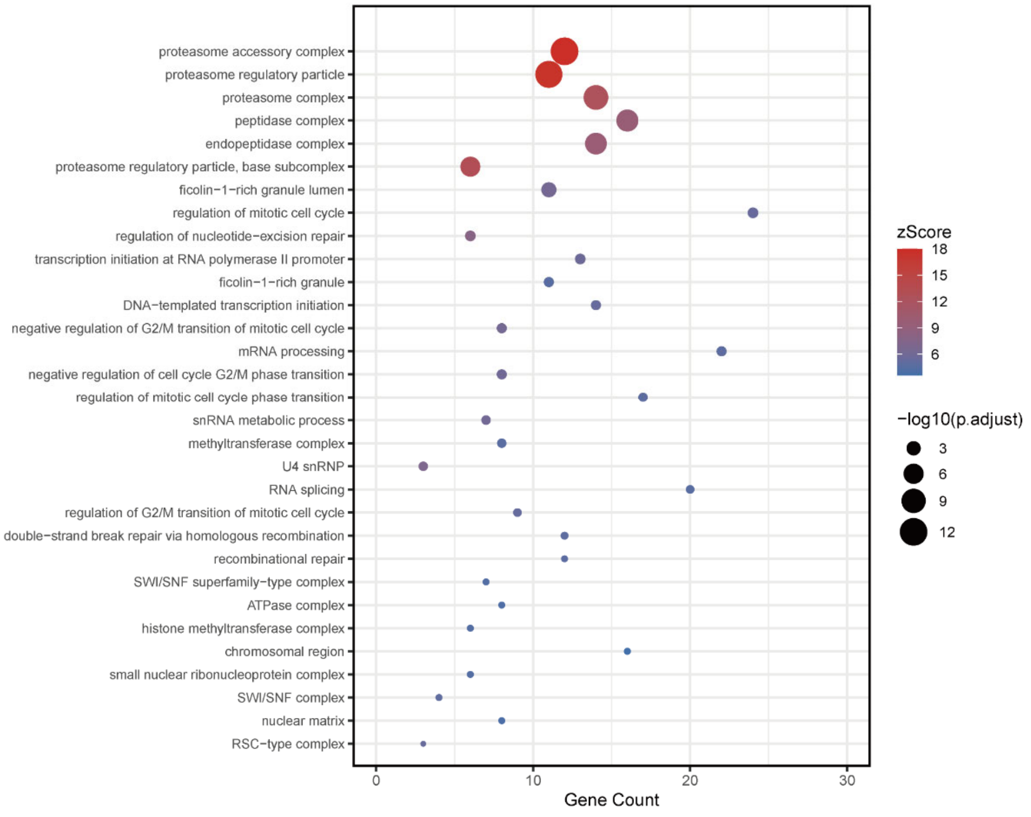

proteasome accessory complex
proteasome regulatory particle
proteasome complex
peptidase complex
endopeptidase complex
proteasome regulatory particle, base subcomplex
ficolin−1−rich granule lumen
regulation of mitotic cell cycle
regulation of nucleotide−excision repair
transcription initiation at RNA polymerase II promoter
ficolin−1−rich granule
DNA−templated transcription initiation
negative regulation of G2/M transition of mitotic cell cycle
mRNA processing
negative regulation of cell cycle G2/M phase transition
regulation of mitotic cell cycle phase transition
snRNA metabolic process
methyltransferase complex
U4 snRNP
RNA splicing
regulation of G2/M transition of mitotic cell cycle
double−strand break repair via homologous recombination
recombinational repair
SWI/SNF superfamily−type complex
ATPase complex
histone methyltransferase complex
chromosomal region
small nuclear ribonucleoprotein complex
SWI/SNF complex
nuclear matrix
RSC−type complex
0
10
20
30
Gene Count
zScore
18
15
12
9
6
−log10(p.adjust)
3
6
9
12

Figure S1. Gene Ontology enrichment analysis of restriction, dependency, and latency-promoting factors. Enrichment analysis was performed using clusterProfiler with the top 300 genes from each phenotype (A) restriction, (B) dependency and (C) latency-promoting against the whole genome as background. Terms were filtered by adjusted P-value < 0.05 and ranked by significance. In each panel, the y-axis lists the enriched GO terms (ordered by adjusted P-value from top to bottom), the x-axis represents the number of genes involved in each term (gene count), the color of the dot indicates the z-score (computed from the enrichment ratio; red: higher z-score, indicating stronger enrichment), and the size of the dot reflects the –log10 (adjusted P-value) (larger dots correspond to more significant terms).

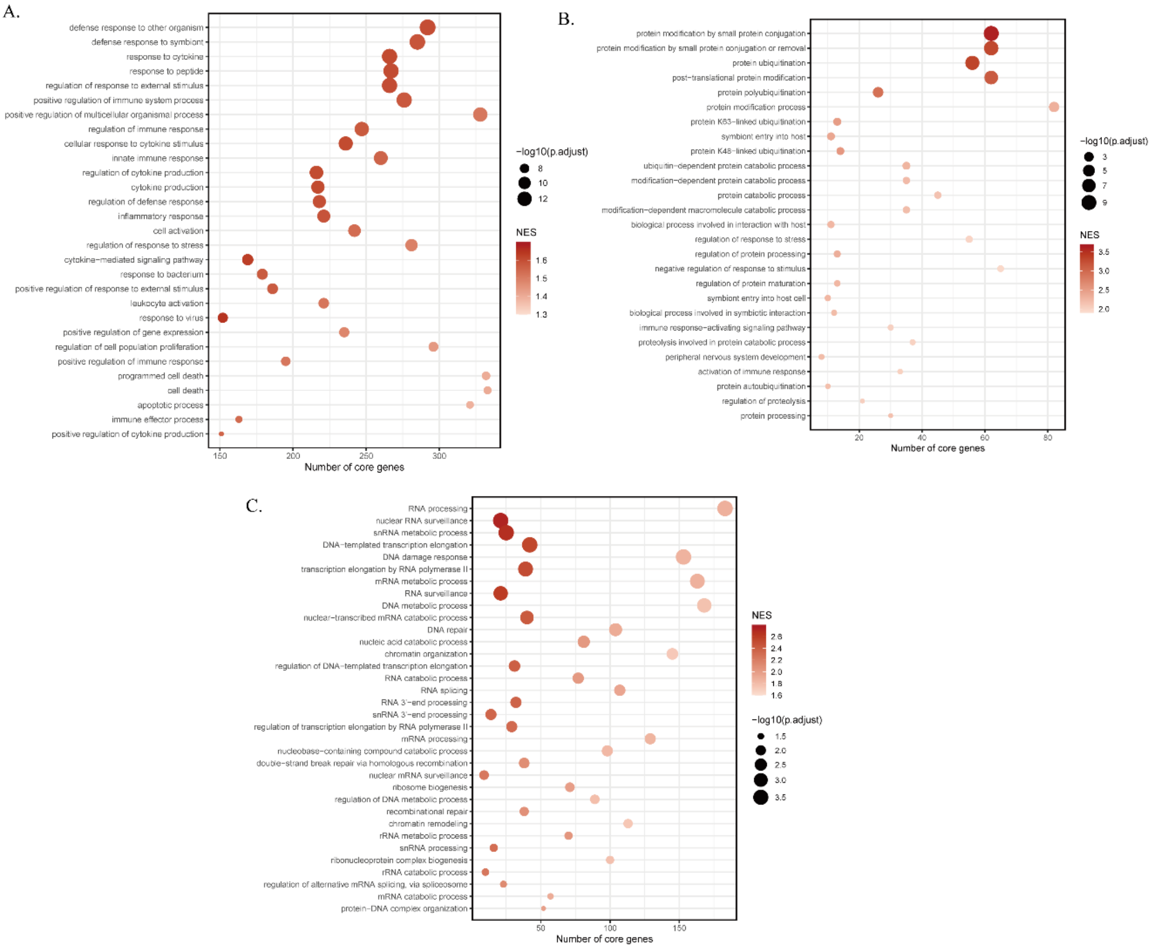

Figure S2. Gene set enrichment analysis (GSEA) of restriction, dependency, and latency-promoting factors. GSEA was performed using the clusterProfiler on ranked lists of genes derived from each phenotype (A) restriction, (B) dependency and (C) latency-promoting. The analysis was restricted to GO Biological Process (BP) terms. The y-axis displays the pathway name (ordered by significance from

top to bottom). The x-axis indicates the number of genes in the core enrichment set. The color of the dot reflects the Normalized Enrichment Score (NES). Red indicates positive enrichment (genes ranked at the top of the list). The size of the dot corresponds to the –log10(adjusted P-value), with larger dots representing more statistically significant enrichments. For restriction phenotype, a total of approximately 490 pathways were significantly enriched (adjusted P-value < 0.05). For clarity, only the top 30 most significantly enriched pathways are shown in panel.
