# Supplementary material for: Systematic Review and Meta-Analysis of Genome-Wide CRISPR-Cas Screens to Identify Host Factors Involved in HIV-1 Infection
Source: Viruses. 2026 Oct 13;18(10):1135. doi: 10.3390/v18101135 (PMC13709478; doi:10.3390/v18101135)
Supplement: Supplementary file 1 [file viruses-18-01135-s001.zip › Supplementary Table S9 - PRISMA checklist.pdf]

PRISMA 2020 Checklist

Section and Topic	Item #	Checklist item	Location where item is reported
**TITLE**			
Title	1	Identify the report as a systematic review.	**Title page**
**ABSTRACT**			
Abstract	2	See the PRISMA 2020 for Abstracts checklist.	**Abstract**
**INTRODUCTION**			
Rationale	3	Describe the rationale for the review in the context of existing knowledge.	**Introduction, paragraphs 1-5**
Objectives	4	Provide an explicit statement of the objective(s) or question(s) the review addresses.	**Introduction, paragraphs 6-7**
**METHODS**			
Eligibility criteria	5	Specify the inclusion and exclusion criteria for the review and how studies were grouped for the syntheses.	**Methods - Data eligibility, Table 1**
Information sources	6	Specify all databases, registers, websites, organisations, reference lists and other sources searched or consulted to identify studies. Specify the date when each source was last searched or consulted.	**Methods - Literature search and study selection**
Search strategy	7	Present the full search strategies for all databases, registers and websites, including any filters and limits used.	**Methods - Literature search and study selection**
Selection process	8	Specify the methods used to decide whether a study met the inclusion criteria of the review, including how many reviewers screened each record and each report retrieved, whether they worked independently, and if applicable, details of automation tools used in the process.	**Methods - Literature search and study selection**
Data collection process	9	Specify the methods used to collect data from reports, including how many reviewers collected data from each report, whether they worked independently, any processes for obtaining or confirming data from study investigators, and if applicable, details of automation tools used in the process.	**Methods - Literature search and study selection**
Data items	10a	List and define all outcomes for which data were sought. Specify whether all results that were compatible with each outcome domain in each study were sought (e.g. for all measures, time points, analyses), and if not, the methods used to decide which results to collect.	**Methods - Gene list extraction and preparation for MAIC**
	10b	List and define all other variables for which data were sought (e.g. participant and intervention characteristics, funding sources). Describe any assumptions made about any missing or unclear information.	**Methods - Gene list extraction and preparation for MAIC**
Study risk of bias assessment	11	Specify the methods used to assess risk of bias in the included studies, including details of the tool(s) used, how many reviewers assessed each study and whether they worked independently, and if applicable, details of automation tools used in the process.	**Not formally assessed. The included studies were gene lists from pooled CRISPR screens rather than interventional studies with effect estimates. Study eligibility was determined by pre-defined criteria (Table 1), and the MAIC algorithm inherently weights genes by the number of independent categories supporting them, which serves as a proxy for cross-study consistency.**
Effect measures	12	Specify for each outcome the effect measure(s) (e.g. risk ratio, mean difference) used in the synthesis or presentation of results.	**Methods - Meta-analysis by information content**
Synthesis methods	13a	Describe the processes used to decide which studies were eligible for each synthesis (e.g. tabulating the study intervention characteristics and comparing against the planned groups for each synthesis (item #5)).	**Methods - Gene list extraction and preparation for MAIC**
	13b	Describe any methods required to prepare the data for presentation or synthesis, such as	**Methods - Gene list extraction and preparation for MAIC**

Section and Topic	Item #	Checklist item	Location where item is reported
		handling of missing summary statistics, or data conversions.	
	13c	Describe any methods used to tabulate or visually display results of individual studies and syntheses.	**Methods - Analysis and visualization of MAIC inputs and results**
	13d	Describe any methods used to synthesize results and provide a rationale for the choice(s). If meta-analysis was performed, describe the model(s), method(s) to identify the presence and extent of statistical heterogeneity, and software package(s) used.	**Methods - Meta-analysis by information content**
	13e	Describe any methods used to explore possible causes of heterogeneity among study results (e.g. subgroup analysis, meta-regression).	**Methods - Meta-analysis by information content**
	13f	Describe any sensitivity analyses conducted to assess robustness of the synthesized results.	Not applicable. Sensitivity analyses were not performed as the primary goal was to integrate ranked gene lists rather than estimate a pooled effect size.
Reporting bias assessment	14	Describe any methods used to assess risk of bias due to missing results in a synthesis (arising from reporting biases).	**Methods - Data eligibility**
Certainty assessment	15	Describe any methods used to assess certainty (or confidence) in the body of evidence for an outcome.	Not applicable. Formal certainty assessment (e.g., GRADE) was not performed as this was an exploratory meta-analysis of ranked gene lists rather than a traditional intervention effect meta-analysis.
**RESULTS**			
Study selection	16a	Describe the results of the search and selection process, from the number of records identified in the search to the number of studies included in the review, ideally using a flow diagram.	**Results - Systematic literature review and study selection**
	16b	Cite studies that might appear to meet the inclusion criteria, but which were excluded, and explain why they were excluded.	**Results - Systematic literature review and study selection**
Study characteristics	17	Cite each included study and present its characteristics.	**Results - Systematic literature review and study selection; Table 2**
Risk of bias in studies	18	Present assessments of risk of bias for each included study.	**Not assessed. The included studies were gene lists from CRISPR screens rather than interventional studies with effect estimates. No widely accepted tool exists for assessing risk of bias in ranked gene lists from functional genomic screens. However, all included studies met pre-defined eligibility criteria, and the MAIC algorithm inherently weights genes by the number of independent publication-level categories supporting them, which serves as a proxy for cross-study consistency.**
Results of individual studies	19	For all outcomes, present, for each study: (a) summary statistics for each group (where appropriate) and (b) an effect estimate and its precision (e.g. confidence/credible interval), ideally using structured tables or plots.	**Results - Overlap among ranked gene lists**
Results of syntheses	20a	For each synthesis, briefly summarise the characteristics and risk of bias among contributing studies.	Study characteristics are summarised in Table 2.Risk of bias was not formally assessed using a standardized tool, as no widely accepted tool exists for evaluating ranked gene lists from CRISPR screens.
	20b	Present results of all statistical syntheses conducted. If meta-analysis was done, present for each the summary estimate and its precision (e.g. confidence/credible interval) and measures of statistical heterogeneity. If comparing groups, describe the direction of the effect.	Results - MAIC analysis of included screens

PRISMA

PRISMA 2020 Checklist

Section and Topic	Item #	Checklist item	Location where item is reported
	20c	Present results of all investigations of possible causes of heterogeneity among study results.	Results - Jaccard similarity
	20d	Present results of all sensitivity analyses conducted to assess the robustness of the synthesized results.	Not formally assessed. The included studies were gene lists from CRISPR screens rather than interventional studies with effect estimates. No widely accepted tool exists for assessing risk of bias in ranked gene lists from functional genomic screens.
Reporting biases	21	Present assessments of risk of bias due to missing results (arising from reporting biases) for each synthesis assessed.	Not formally assessed. Studies lacking accessible gene lists or raw data were excluded during study selection (see Methods - Data eligibility), which reduced the risk of reporting bias.
Certainty of evidence	22	Present assessments of certainty (or confidence) in the body of evidence for each outcome assessed.	Not applicable. Formal certainty assessment (e.g., GRADE) was not performed as this was an exploratory meta-analysis of ranked gene lists rather than a traditional intervention effect meta-analysis.
**DISCUSSION**			
Discussion	23a	Provide a general interpretation of the results in the context of other evidence.	Discussion
	23b	Discuss any limitations of the evidence included in the review.	Discussion - Limitations
	23c	Discuss any limitations of the review processes used.	Discussion - Limitations
	23d	Discuss implications of the results for practice, policy, and future research.	Discussion - Conclusions
**OTHER INFORMATION**			
Registration and protocol	24a	Provide registration information for the review, including register name and registration number, or state that the review was not registered.	This systematic review was registered in PROSPERO (CRD420261438705)
	24b	Indicate where the review protocol can be accessed, or state that a protocol was not prepared.	No protocol was prepared
	24c	Describe and explain any amendments to information provided at registration or in the protocol.	Not applicable.
Support	25	Describe sources of financial or non-financial support for the review, and the role of the funders or sponsors in the review.	The work was supported by the Guidance Program for the Agricultural and Social Development Research of Hangzhou (grant number 20241029Y081), the Zhejiang Provincial Science and Technology Program for Disease Prevention and Control (grant number 2025JK043), the Zhejiang Provincial Key Laboratory Construction Project (2024ZY01026), and the Construction Fund of Key Medical Disciplines of Hangzhou (2025HZGF13)
Competing interests	26	Declare any competing interests of review authors.	The authors declare no conflicts of interest
Availability of data, code and other materials	27	Report which of the following are publicly available and where they can be found: template data collection forms; data extracted from included studies; data used for all analyses; analytic code; any other materials used in the review.	All data generated or analysed during this study are included in the published article and its supplementary information files. The custom R scripts for the MAIC analysis are openly available in the GitHub repository: https://github.com/Min123-xu/HIV-MAIC-host-factors.

From: Page MJ, McKenzie JE, Bossuyt PM, Boutron I, Hoffmann TC, Mulrow CD, et al. The PRISMA 2020 statement: an updated guideline for reporting systematic reviews. BMJ 2021;372:n71. doi: 10.1136/bmj.n71. This work is licensed under CC BY 4.0. To view a copy of this license, visit https://creativecommons.org/licenses/by/4.0/
